# The safety and usefulness of neutron brachytherapy and external beam radiation in the treatment of patients with gastroesophageal junction adenocarcinoma with or without chemotherapy

**DOI:** 10.1186/1748-717X-9-99

**Published:** 2014-04-29

**Authors:** Qifeng Wang, Tao Li, Huiming Liu, Xitang Jia, Bo Liu, Xin Wan, Jinyi Lang

**Affiliations:** 1 Department of Radiation Oncology, Sichuan Cancer Hospital, Chengdu 610041, People’s Republic of China; 2 Department of Radiation Oncology, Changzhi Cancer Hospital, Changzhi 046000, People’s Republic of China; 3 Department of Radiation Oncology, Fouth Hospital of Hebei Medical University, Shijiazhuan 050011, People’s Republic of China

**Keywords:** Gastroesophageal junction adenocarcinoma (GEJAC), Neutron brachytherapy (NBT), External beam radiation (EBRT), Overall survival acute/late toxicity

## Abstract

**Purpose:**

To assess the safety and usefulness of neutron brachytherapy (NBT) as an adjuvant in the treatment of patients with gastroesophageal junction adenocarcinoma (GEJAC) with external beam radiation (EBRT), with or without chemotherapy.

**Methods and Materials:**

In total, 197 patients with localized, advanced GEJAC received EBRT and NBT with or without chemotherapy. Radiotherapy consisted of external irradiation to a total dose of 40–54 Gy (median 50 Gy) and brachytherapy to 8–25 Gy (median 20 Gy) in two to five fractions. In total, 88 patients received chemotherapy that consisted of two cycles of a regimen with CDDP and 5FU from days l-4. The cycles were administered on days 1 and 29. MMC was given alone in bolus injection on day 1 each week. The cycles were administered on days 1, 8, 15 and 22.

**Results:**

The duration of follow-up ranged from six to 106 months (median 30.4 months). The median survival time for the 197 patients was 13.3 months, and the one, two, three- and five-year rates for overall survival were 57.1%, 35.1%, 23.0% and 9.2%, respectively. For acute toxicity, no incidences of fistula and massive bleeding were observed during this treatment period. In total, 159 (80.7%) patients developed Grade 2 hematologic toxicity and 146 (74.1%) patients developed Grade ≥ 2 esophagitis. The median times of incidence of fistula and bleeding were 9.5 (3–27.3) months and 12.7 (5–43.4) months, respectively. The incidence of severe, late complications was related to higher NBT dose/f (20–25 Gy/5 F) and higher total dose(≥70 Gy). In total, 75.2% of the patients resumed normal swallowing and 2.0% had some residual dysphagia (non-malignant) requiring intermittent dilatation.

**Conclusion:**

A combination of EBRT and NBT with the balloon type applicator was feasible and well tolerated. Better local-regional control and overall survival cannot achieved by a higher dose, and in contrast, a higher dose caused more severe esophageal injury.

## Introduction

Tumors of the lower esophagus and the proximal stomach are usually classified as gastroesophageal junction adenocarcinomas (GEJAC). These carcinomas are the most rapidly increasing type of tumor in many Western countries, and they represent an aggressive disease with poor prognosis [1,2]. With the present state of knowledge, some patients with localized GEJ cancers are treated with surgery alone; others with chemotherapy pre- and postoperatively; and some with trimodality therapy, either preoperatively or postoperatively. The choice of treatment mostly depends on the preferences of the treating team of physicians.

The nonsurgical management of patients with GEJAC, including the use of laser coagulation or self-expanding metallic stents with radiation, has been considered for decades to only be a palliative modality. However, for patients with inoperable GEJAC or those who have rejected an operation, there has yet to be any studies on the safety and usefulness of neutron brachytherapy (NBT) in their treatment.

Californium-252 (^252^Cf) is a neutron-emitting radionuclide, and ^252^Cf-based NBT has only been implemented in China very recently [3]. NBT is a form of high linear energy transfer (LET) radiotherapy, which has been proven to be effective for treating intracavitary cancers of the cervix when used in combination with external beam radiotherapy (EBRT) [4,5]. However, no studies regarding the treatment of GEJAC by EBRT combined with NBT have been reported. NBT is thought to be a viable option for treating GEJAC for at least three reasons. First, GEJAC is resistant to the conventional, low-LET X-ray or gamma-ray radiotherapy [6]. NBT is a form of high-LET radiotherapy, which has been shown to be effective in killing radio-resistant cancer cells [5,7]. Second, the location of GEJAC makes it easily accessible to the ^252^Cf neutron source via the use of an applicator/catheter. Third, water is an effective neutron shield, and it can be injected into the source applicator during treatment to reduce the neutron dose to the normal tissue near the tumor.

The aim of the present study was to observe and analyze the long-term curative effects and complications of NBT as an adjuvant in the treatment of patients with GEJAC with EBRT with or without chemotherapy.

## Materials and methods

### Patients

From Jan 2001 until November 2012, a total of 197 consecutive patients with localized, advanced gastroesophageal cancer were referred to our department at the Changzhi Cancer Hospital for radiotherapy and ^252^Cf NBT. The reasons were as follows: 10 patients were medically inoperable; 40 rejected surgery; 83 were too old (71 years or older, 34 of 83 had T4 lesion); 93 had unresectable lesions and one had other malignancy. Of these 197 patients, 88 were treated with chemoradiotherapy combined with brachytherapy (the CRT group). Patients with good performance status (at least able to care for himself or herself) and adequate hepatic, renal, and hematologic functions were selected for curative treatment. All of the patients had adenocarcinoma. The patients’ 6th AJCC stages were diagnosed to be Stage II to III by barium examination, endoscopy, endoscopic ultrasonography or tumor histology. The classification of GEJAC was divided into Type I to Type III [8]. All of the patients gave their informed consent before treatment, which was in accordance with the Declaration of Helsinki and also approved by the Ethics Committee of Changzhi Cancer Hospital. The demographic data and tumor characteristics of each group are given in Table 1.

**Table 1:** Patient/tumor characteristics

**Characteristics**	**Total (%)**	**CRT (%)**	**RT (%)**	***p *** **Value**
Sex				0.008
Male	153 (77.7)	76 (86.4)	77 (70.6)	
Female	44 (22.3)	12 (13.6)	32 (29.4)	
Age (years)				0.028
≤65	73 (37.3)	40 (45.5)	33 (30.3)	
>65	124 (62.7)	48 (54.5)	76 (69.7)	
KPS				0.141
≥80	114 (57.9)	56 (63.6)	58 (53.2)	
70	83 (42.1)	32 (36.4)	51 (46.8)	
The length				0.358
≤5 cm	182 (92.4)	83 (94.3)	99 (90.8)	
>5 cm	15 (7.6)	5 (5.7)	10 (9.2)	
Tumor location				0.092
Type I	9 (4.6)	4 (4.5)	5 (4.6)	
Type II	95 (48.2)	35 (36.8)	60 (55.0)	
Type III	93 (47.2)	49 (55.7)	44 (40.4)	
T stage				0.002
T2	36 (18.3)	8 (9.1)	28 (25.7)	
T3	68 (34.5)	31 (35.2)	37 (33.9)	
T4	93 (47.2)	49 (55.7)	44 (40.4)	
N stage				<0.0001
N0	49 (24.9)	9 (10.2)	40 (36.7)	
N1	148 (75.1)	79 (89.8)	69 (63.3)	
6th AJCC stage				<0.0001
II	46 (23.4)	9 (10.2)	37 (33.9)	
III	151 (76.6)	79 (89.8)	72 (66.1)	
Total dose				0.235
≤70	67 (34.0)	26 (29.5)	41 (37.6%)	
>70	130 (66.0)	62 (70.5)	68 (62.4)	

*Abbreviations*: *CRT * chemotherapy plus radiotherapy, Total dose EBRT + NBT dose, *OS* Overall survival rate, *LRC* Local-regional control.

### Radiotherapy

Megavoltage radiation therapy units were used with a minimum source-to-axis distance of 100 cm. The radiation field extended at least 3 cm superior and inferior to the tumor, with a lateral margin of at least 2 cm. The field included the lesser curvature and bottom of stomach if the tumor was type III. The boost radiation field was the same length. Multi-field techniques were used to limit the maximum dose to the spinal cord to ≤45 Gy. The radiation treatments were delivered five days/week and at 2 Gy/fraction. The initial anterior-posterior parallel-opposed fields received 30 Gy and the off-cord fields received 20–30 Gy, for a total dose of 40–54 Gy in 20–27 fractions in 4–5.5 weeks.

**Figure 1:**
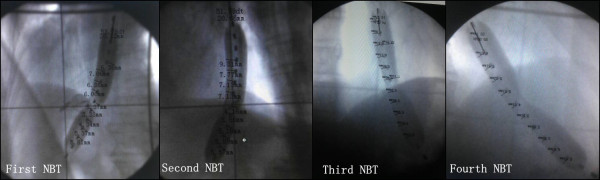
**An X-ray image was taken while the applicator and the source were both inserted into the esophagus of a patient.** The water balloon can clearly be seen as it is filled with an X-ray contrast agent. The dose was prescribed to the reference point, which was located 10 mm from the center point of the source capsule in the transverse direction. From the first to fourth NBT, the shape of the tumor can be clearly identified.

NBT with a one-balloon applicator (Figure 1) was used in conjunction with the ^252^Cf LZH-1000 remote after-loading system (Linden Science and Technology Co, Shenzhen, China). The physical characteristics of ^252^Cf Neutron, the characteristics of the applicator and the process of NBT were described in detail by Liu H [9]. The dose was prescribed to the reference point, which was located at 10 mm from the center point of the source capsule in the transverse direction. Figure 1 is an X-ray image taken while the applicator and the source were both inserted into the esophagus of a patient. In Figure 1, the water balloon can clearly be seen as it is filled with an X-ray contrast agent. The dose was prescribed to the reference point, which located 10 mm from the center point of the source capsule in the transverse direction. The total radiation dose (to the reference point) given to each patient varied between 8–25 Gy-eq in two to five fractions, with 4–5 Gy-eq per fraction per week.

### Chemotherapy

Chemotherapy consisted of three cycles of a regimen with CDDP (20 mg/m^2^/d in 2 h infusion) and 5FU (500 mg/m^2^/d in continuous infusion) from days l-4. The cycles were administered on days 1 and 29. MMC was given alone in a bolus injection on day 1 per week at 4 mg/m^2^. The cycles were administered on days 1, 8,15 and 22.

### Toxicity assessment and follow-up

The patients were examined weekly during the course of external beam radiation. Weekly blood tests were obtained, and any admission for treatment-related complications was recorded. All adverse events were graded according to the National Cancer Institutes Common Terminology Criteria for Adverse Events, version 3.0 [10].

The patients usually underwent follow-up examinations every 3–4 months after the completion of treatment. Tumor response and nodal disease were evaluated with repeated CT scans, barium swallow studies and endoscopy.

### Statistical analysis

The objectives of the study were to evaluate overall acute toxicity and local-regional control rates. Death from esophageal cancer was considered as treatment failure in the survival analysis. Survival was calculated from the date of consultation until death or last follow-up evaluation. The pattern of failure (local and/or regional vs. distant) was defined as the first site of failure. The time to first failure, time to any local failure and time to any distant metastases were calculated from the date of consultation. Local and regional recurrence included the primary tumor and regional lymph nodes. Overall survival and local-regional control were estimated using the Kaplan–Meier method. Pearson’s chi-square test was used to assess measures of association in the frequency data. A value of p<0.05 was considered statistically significant.

## Results

### Patients’ characteristics and treatments

The ages of the esophageal cancer patients who were treated with radiation therapy (NBT and EBRT) ranged from 44 to 84 years (median, 69 years). The tumor stage was distributed as follows for the 197 patients: II (n = 46) and III (n =151) by 6th AJCC tumor stage. All patients completed the planned EBRT + NBT treatment. Among these patients, 88 patient received CRT, of which 25 and 63 patients received the PF and MMC alone regimens, respectively. The patients’ characteristics and treatments are summarized in Table 1.

### Prognostic factors for overall survival and local-regional control

The duration of follow-up ranged from six to 106 months (median 30.4 months). The median survival time for the 197 patients was 13.3 months, and the one-, two-, three- and five-year rates for OS were 57.1%, 35.1%, 23.0% and 9.2%, respectively. The one-, two-, three- and five-year rates for LRC were 76.6%, 61.5%, 50.1% and 35.9%, respectively.

**Figure 2:**
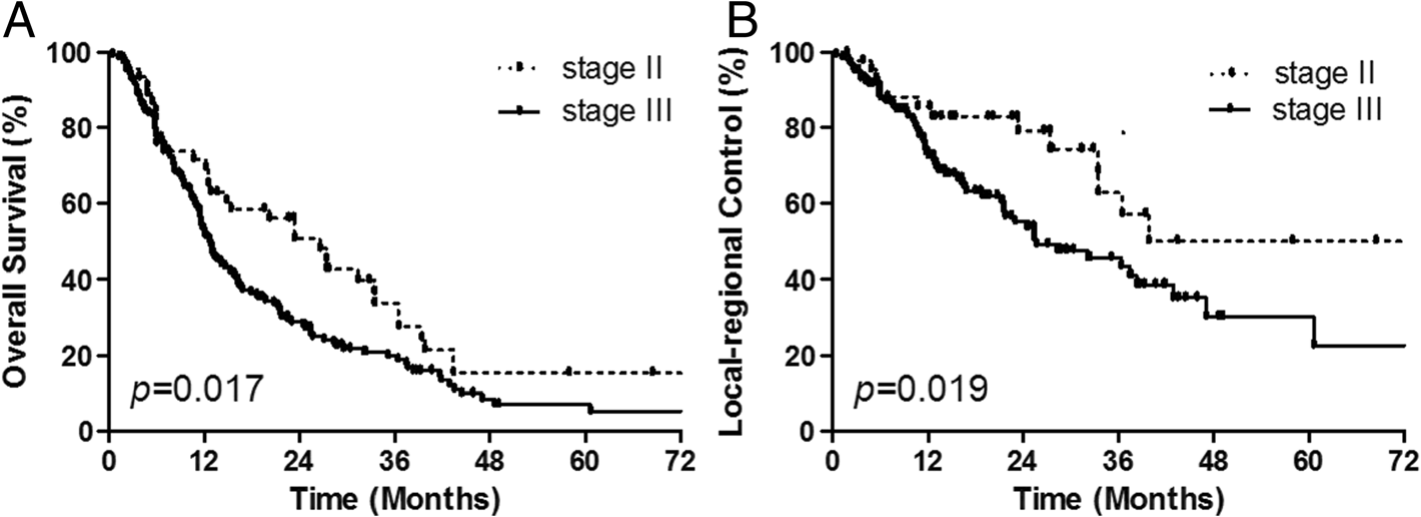
**Comparison of overall survival (OS, A) and local-regional control (LRC, B) between patients with different clinical stages.** Statistically significant differences were found in LRC and OS, which favored stage II patients.

We used the nine following factors for the univariate analysis of survival rates and local control rate: sex, age, tumor location, tumor length, tumor T stage, nodal stage, clinical stage, concurrent chemotherapy, and radiation dose. We found that clinical stage was the only factor that was significantly related to OS and LRC (Figure 2, *p* = 0.017 and *p* = 0.019, respectively). In the univariate analysis, the five-year OS (LRC) was 15.3% (50.1%) and 7.0% (30.2%) for stage II and III group patients, respectively (*p* = 0.017, *p* = 0.019). We did not observe that the CRT regimen and increasing the total dose was able to significantly increase the OS and LRC of patients.

### Patterns of failure

At the time of the analysis, 28 patients were alive and free of disease and five patients were alive with disease evolution. Distant metastases occurred in 40 patients (20.3%). The median time to developing distant metastases was 13.3 months. The main sites of distant metastases were the lung (n = 13), liver (n = 5), brain (n = 4) and bone(n = 4). In 14 patients, metastases developed in more than one organ. Two patients developed second primary tumors. Additionally, 37 patients died of mixed reasons, including pneumonia, cerebral hemorrhage, heart infarction and a car accident. Local-regional recurrence occurred in 76 (76/197,38.6%) patients, with 44/76 (57.9%) occurrences outside the radiation fields and 58/76 (76.3%) occurrences inside the radiation fields. Additionally, 26/76 (34.2%) had both inside and outside filed recurrences. None of those patients underwent salvage surgery.

### Treatment toxicity

All 197 patients completed the planned NBT and EBRT treatment. In terms of acute toxicity, no perforations were observed during this treatment period. In total, 159 (80.7%) patients developed a Grade 2 hematologic toxicity. Dysphagia was relieved after the second or third NBT treatment in 95% of the patients, and a temporary feeding tube was not required in most of the patients. Grade ≥ 2 esophagitis, expressed by clinical odynophagia, was observed in 146 cases (74.1%) and was managed with the early introduction of H2 blockers and surface anesthesia at the initiation of the NBT. In total, 12 patients had Grade ≥ 2 irradiation dermatitis. From the time of treatment completion to the development of local-regional recurrence or death at the follow time, 8(4.1%) and 13 (6.6%) patients experienced fistula and massive bleeding, respectively. The median time of incidence of fistula and bleeding was 9.5(3–27.3) months and 12.7(5–43.4) months, respectively. As shown in Table 2, the incidence of severe, late complications was related to higher NBT dose/f (20–25 Gy/5 F) and higher total dose(≥70 Gy). In total, 75.2% of the patients resumed normal swallowing, while 2.0% had some residual dysphagia (non-malignant) requiring intermittent dilatation. For acute toxicity, CRT increased the incidence of Grade ≥ 2 pneumonia more than RT (11.0% vs. 2.3%, p = 0.018). Other acute toxicities and late complications did not have any significant relationship to higher total dose and receiving the CRT regimen.

**Table 2:** shows the relationship between NBT and EBRT dose factor and severe complications

**Factors**		**Fistula**	**Bleeding**
CRT	Yes	4	4
	No	4	9
NBT dose/F	12 Gy/3 F	2	1
	16 Gy/4 F	1	2
	20 Gy/5 F	4	7
	25 Gy/5 F	1	3
EBRT	40 Gy	2	2
	50 Gy	6	9
	52 Gy		1
	60 Gy		1
Total dose	52-56 Gy	2	1
	60-66 Gy	1	2
	70-75 Gy	5	10

Note: CRT = chemotherapy plus radiotherapy, Total dose = EBRT + NBT dose.

F = fraction, NBT = neutron brachytherapy, EBRT = external beam radiotherapy.

## Discussion

Preoperative chemotherapy and preoperative chemoradiotherapy followed by surgery are well established in the curative treatment of patients with localized GEJAC [11,12]. However, for patients with locally advanced, non-resectable and inoperable GEJAC, palliative therapy, including the use of intraluminal stents, photodynamic therapy, brachytherapy and argon plasma coagulation [13], is the main choice. The use of stents has changed clinical practice in patients with critical dysphagia [14], and a recent trial supports the continued use of palliative radiotherapy, as it confers benefits on patient survival and quality of life. There is randomized trial evidence that single dose, intraluminal brachytherapy provides better long-term relief of dysphagia with improved quality of life than stents but requires a longer time to symptomatic relief [15]. However, a few studies have reported the safety and inefficacy of external radiotherapy and brachytherapy as a curative treatment for GEJAC. We retrospectively analyzed our database and found that first, using NBT as an adjuvant in the treatment of patients with GEJAC with EBRT with or without chemotherapy is well tolerated and useful. Second, improving the total dose and combining it with chemotherapy does not result in better OS and LRC and also causes a higher incidence of late, severe complications. Third, for patients with stage II, this treatment can result in improved OS.

Prior to this report, the number of studies using EBRT and BT to boost concurrent chemotherapy in a meaningful number of patients have been limited [16]. Table 3 summarizes these experiences, as well as our own. The present study shows survival benefits for addition of NBT to EBRT for treating locally advanced disease, resulting in a median overall survival time of 13.3 months, as Hishikawa et al. [17] and Hareyama et al. [18] have previously reported.

**Table 3:** Clinical results of external beam radiation, brachytherapy boost, with or without chemotherapy

**Authors (Ref.)**	**Hishikawa et al. **[17]	**Flores et al. **[23]	**Hareyama et al. **[18]	**Sharma et al. **[25]	**RTOG9207 **[16]	Present study*
No. of pts.	148	171	161	100	50	197
BT Gy/fraction	12/1	15/1	15-20/NS	15-20/1	15/3	12-25/2-6
EBRTGy/fraction	60/30	40/15	47-70/25-35	50/28	50	30-60
CT (pts)	No	No	No	Yes	Yes	88/197
Fistula (%)	5.3	5	1.2	12	12	8 (4.1%)
Bleeding (%)	0%	11	0	4	NS	13 (6.6%)
Ulcer (%)	NS	NS	3	29	NS	NS
Stricture (%)	10	35	3	16	4	4 (2%)
Death rate (%)	3	0.6	0	4	8	0
OS (%)	37 (2 y) LD	33 (1 y)	43.3/Stage I,	8 (20 Gy)	49 (1 y)	57.1 (1 y)
	8 (2 y) ED	26 (2 y)	21.1/Stage II,	23 (15 Gy)		35.1 (2 y)
		19 (3 y)	(5 y)	(5 y)		9.2 (5 y)
LRC (%)	64 (2 y) LD	NS	31.7%(5 y)	NS		35.9 (5 y)

*Abbreviations:CT* Chemotherapy, *HDR* high dose rate, *NS* not stated, *LD* limited disease, *ED* extensive disease, *y* year.

The optimal dose for using BT as an adjuvant is unknown, but the incidence of late complications has been related to the dose to the mucosa [19], dose per fraction [20,21], dose rate [22] and chemotherapy [23]. Sur et al. [24] observed a difference in the local control that depended on whether the boost was delivered with 20 Gy or 12 Gy HDRBT after an initial 35 Gy EBRT. At one year, the local control was, 25% and 70.6%, respectively. In the present study, the boost dose in patients varied from 20 Gy to 25 Gy, with the total dose being less than 70 Gy. These doses did not significantly prolong the patients’ OS or improve their LRC. Additionally, for GEJAC, blindly increasing the RT dose did not increase the OS and LRC, and further research should include studies on the reasonable dosage scope or the combination with other treatments, such as chemotherapy or targeted therapy, to improve curative effect.

The risk of late complications seems to be strongly affected by a higher dose and a large fraction size of HDR BT [16,25]. Sharma compared the treatment-related complications in groups 1 (20 Gy BT boost) and 2 (15 Gy BT boost) and reported strictures in 24% vs. 8% (p = 0.029), respectively, ulceration in 30% vs. 28% (p = 0.8), respectively, and tracheoesophageal fistulae in 12% of patients in both groups [25]. A high incidence of esophageal fistulas (8%) was reported in the RTOG 9207 study [16]. In this study, a dose of 8–25 Gy in 2–5 fractions via HDR NBT was delivered. Our study also demonstrated that 21 patients treated with EBRT and NBT developed severe, late toxicity. The incidence of late, severe complications was significantly related to higher total dose and NBT dose factors. In addition to dose factors, the combined treatment with chemotherapy also significantly increased the incidence of relevant, late complications. Atsunori Yorozu reported that treatment-related esophageal ulceration or strictures occurred in 18 patients (34%) in the CRT group, compared with 12% in the RT group (p = 0.013) [26]. RTOG 9207 [16] documented treatment-related esophageal fistulas in 12% of the patients. In comparison, none were reported in several other BT and EBRT without CT series [17,18,23,27]. The present study has documented no acute fistulas of the patients, compared to the reports by Hishikawa et al. [17] or Gava et al. [27]. However, the direct comparison of these clinical series is hampered by the differences in staging, classification, response end points and duration of follow-up. Table 3 shows that NBT + EBRT with or without CT resulted in treatment complication similar to that which has been reported [17,18,23,25,27]. Because ^252^Cf NBT is a form of high-LET radiotherapy, we believe that it is superior to conventional X-ray radiotherapy in treating radio-resistant esophageal cancers. An additional advantage of NBT over X-ray radiotherapy is the fact that water can be injected into the source applicator during treatment to reduce the neutron dose to nearby normal tissue. RTOG 9207 [16] reported that increased courses of chemotherapy and chemotherapy concurrent with brachytherapy may significantly improve the incidence of late, severe complications. In the present study, the incidence of severe, late complications in the CRT group was similar to that in the RT alone group. This can be explained by several main reasons. Firstly, the chemotherapy regimens were multifarious, with most of the patients receiving the MMC alone regimen. Secondly, the concurrent chemotherapy doses were lower than those used in normal chemotherapy alone regimens and may have resulted in radiotherapy sensitization. Thirdly, neutron irradiation overcomes the radiation resistance of the tumor, thus reducing the chemotherapy sensitization effect.

## Conclusion

In summary, NBT + EBRT is a highly effective and well-tolerated therapeutic modality, which can be used not only as a palliative therapy but also as a radical treatment for patients with inoperable GEJAC. Statistically significant better LRC and OS were observed in patients with stage II disease. Increasing the total dose and combining it with chemotherapy did not improve OS and LRC and resulted in a higher incidence of late, severe complications. However, the accuracy of the data on toxicity was limited by the retrospective nature of this study. Methods to reduce treatment toxicity and increase the tumor response to radiotherapy, thereby increasing the therapeutic ratio, are needed. Our data indicate a need for additional studies on the optimal EBRT and NBT dose in a prospective randomized trial. In particular, the studies should place emphasis on decreasing the treatment-related toxicity of concurrent chemoradiotherapy, as it is the only treatment modality available for patients with locally advanced GEJAC.

## Competing interests

The authors declare that they have no competing interests.

## Authors’ contributions

QW and HL carried out data acquisition, performed the statistical analysis, drafted the manuscript and participated in the sequence alignment. JL and TL conceived of the study, participated in the design of the study. XJ, XW and BL participated in the sequence alignment. QW, HL and TL participated in its design and coordination and helped to draft the manuscript. All authors read and approved the final manuscript.
